# Modelling the Transmission Dynamics and Risk of Ovine Cystic Echinococcosis in Argentina Using the CEST Model (Cystic Echinococcosis Stochastic Transmission Model)

**DOI:** 10.1155/japr/6394607

**Published:** 2026-09-10

**Authors:** Jo Widdicombe, Mahbod Entezami, María-Gloria Basáñez, Daniel Jackson, Edmundo J. Larrieu, Guillermo Mujica, Joaquín M. Prada

**Affiliations:** ^1^ Department of Comparative Biomedical Sciences, School of Veterinary Medicine, Faculty of Health and Medical Sciences, University of Surrey, Guildford, UK, surrey.ac.uk; ^2^ MRC Centre for Global Infectious Disease Analysis and London Centre for Neglected Tropical Disease Research, Department of Infectious Disease Epidemiology, School of Public Health, Imperial College London, London, UK, imperial.ac.uk; ^3^ Surrey Health Economics Centre, School of Biosciences, Faculty of Health and Medical Sciences, School of Biosciences, University of Surrey, Guildford, UK, surrey.ac.uk; ^4^ School of Veterinary Medicine (Escuela de Veterinaria y Producción Agroindustrial), Universidad Nacional de Río Negro, Choele Choel, Río Negro, Argentina, unrn.edu.ar; ^5^ Veterinary Public Health, URESA Andina, Ministry of Health of Río Negro, Viedma, Río Negro, Argentina

## Abstract

**Background:**

Cystic echinococcosis (CE) is a neglected zoonotic disease caused by *Echinococcus granulosus sensu lato* (s.l.). Exerting substantial health and economic impact on humans, and the agricultural industry of affected countries, CE is one of 20 neglected tropical diseases included in the 2021–2030 World Health Organization (WHO) roadmap to attain the United Nations Sustainable Development Goals.

**Methods:**

The CEST model, a stochastic disease transmission model for CE, captures the lifecycle and disease dynamics of the parasite in dogs and sheep. Calibrated with data from Río Negro, Argentina, and incorporating annual fluctuations in livestock husbandry, the model includes historical interventions employed in the Río Negro control programme since its onset (1980) in a retrospective analysis. A prospective analysis models various intervention scenarios (2025–2035). Dog deworming with praziquantel and sheep vaccination with EG95 are simulated at different frequencies and coverage levels. Annual CE prevalence in dogs, sheep and predicted human risk are presented for each intervention.

**Results:**

Monthly deworming most rapidly reduced dog prevalence and environmental egg burden; however, increasing vaccine coverage from 60% to 80% achieved a greater impact on sheep prevalence. Vaccination was less effective at 60% coverage, highlighting the importance of achieving the higher coverage and full dosing of animals in control programmes. Although current interventions can reduce disease prevalence in dogs and sheep, even when they are modelled at increased frequencies and coverage, sporadic transmission is predicted to continue. To break the status quo, alternative intervention packages need to be considered.

**Conclusions:**

Enhancing CE control requires additional measures, including a deeper understanding of factors contributing to disease persistence despite current control efforts. The CEST model can be extended to simulate additional interventions, making it a valuable tool for policymakers and stakeholders in planning CE control efforts beyond the Sustainable Development Goals.

## 1. Introduction

Cystic echinococcosis (CE) is a neglected zoonotic disease caused by the cestode *Echinococcus granulosus sensu lato* (s.l.). The parasite is distributed globally, with regions of high endemicity of human CE infection in the Mediterranean littoral, Asia, the Middle East including Afghanistan and Pakistan, and South America [1]. CE exerts a substantial health and economic impact on humans and the agricultural industry, causing high morbidity and mortality among affected human populations [2]. The Global Burden of Disease (GBD) study estimated that CE was responsible for 1350 deaths (95% uncertainty interval (UI) = 987–1760) and 122,000 (95% UI = 89,200–169,000) disability‐adjusted life years (DALYs) in 2019. The global estimate of cases was 0.9 (0.6–1.4) million. In central Asia, CE was the neglected tropical disease (NTD) with the greatest burden, with 30,100 DALYs (95% UI = 15,300–54,400) in 2019 [3]. Consequently, CE is one of 20 NTDs prioritised in the 2021–2030 World Health Organization (WHO) Roadmap to end their neglect and attain the United Nations Sustainable Development Goals. Specifically, CE is targeted for intensified control in hyperendemic areas of 17 countries by 2030 [4].

*E. granulosus* is a parasite of the Taeniidae family, which requires two mammalian hosts to complete its lifecycle. The most widespread transmission occurs between dogs and sheep; however, humans can become accidentally infected and be dead‐end hosts [5]. Infected dogs are the definitive hosts, harbouring adult worms in their small intestines and passing out parasite eggs in their faeces. The eggs contaminate the environment and can be subsequently ingested by the intermediate host (sheep) when they graze in contaminated pastures. After ingestion, the eggs hatch and release embryos (oncospheres) in the intestines of the intermediate host. Penetration of the intestinal mucosa leads to the haematogenous spread of the oncospheres to the liver and other body organs, where larval development begins and cystic structures form. Ingestion of the cyst‐containing offal by dogs, either via scavenging of deadstock or through deliberate offal feeding, leads to reinfection of the definitive host and completion of the parasite lifecycle [6].

Human CE, also known as hydatid disease, occurs from the accidental ingestion of eggs from the environment, either through hand to mouth contact or contaminated food and water. This leads to the formation of hydatid cysts within organs such as the liver and lungs [7, 8]. Disease progression is unpredictable; although people may remain asymptomatic for years, if left untreated, the cysts may grow to a considerable size requiring surgical intervention. In rare circumstances morality may occur ([9, 10]).

Although a range of potential intervention strategies exist for the control of CE in its zoonotic hosts, including anthelmintic treatments and prophylactic vaccination, there are very few countries in which elimination has been achieved. In some South American countries, CE surveillance and control programmes are absent or in their infancy. In other countries such as Argentina, regional surveillance programmes exist. The control programme in Río Negro, a province in northern Patagonian Argentina, is the oldest, having started in 1980 and implemented continuously thereafter. Despite a considerable reduction in ovine and canine CE prevalence, transmission still occurs within the province. This necessitates further understanding of disease dynamics and the evaluation of various intervention strategies that could support the programme.

Mathematical transmission dynamics models are increasingly being utilised to evaluate control strategies for parasitic diseases and as a tool for dynamic disease risk projection [11]. Such models can potentially add value to pilot studies and existing control programmes by generating projections of the impact of various intervention strategies, alone or in combination, to control or eliminate a disease [12, 13]. In addition to projecting the time span in which control or disruption of disease transmission could be achieved, the impact of programme interruptions, such as those during the SARS‐CoV‐2 (COVID‐19) pandemic, can also be investigated, as was done for other NTDs [14].

Several models have been published for CE since the 1980s, providing a descriptive framework of the disease in countries such as China and Kazakhstan [15–23]. The majority of existing CE models are force of infection, integro‐differential equation and compartmental models which simulate parasite dynamics at the population level. However, there are also two individual‐based (or agent‐based) models, representing populations as collections of discrete individuals that can have heterogenic attributes such as spatial location, physiological traits and behaviours applied [24–26].

However, none have been developed specifically for South America in general or Argentina in particular to capture the unique characteristics of their farming systems. Data‐informed models are more precise in their predictive performance and estimated timelines for elimination; however, all model projections are subject to substantial uncertainty dependent on the reliability of information upon which they are based and evaluated against [13]. Stochastic models that incorporate parameter uncertainty can produce model outcomes which fall within a range [27], reflecting the complexity and variability of the input parameters. The limited use of stochastic models for CE in the South America region represents a critical research gap. Thus, to reflect upon and inform the Argentinian CE control programme, as well as other programmes in Latin America, there is a need for a model with parameters and initial conditions informed by regional data. Here, a model based on the farming system in Río Negro is presented, with projections of transmission dynamics given the implementation of different intervention scenarios.

## 2. Model and Methods

**Figure 1:**
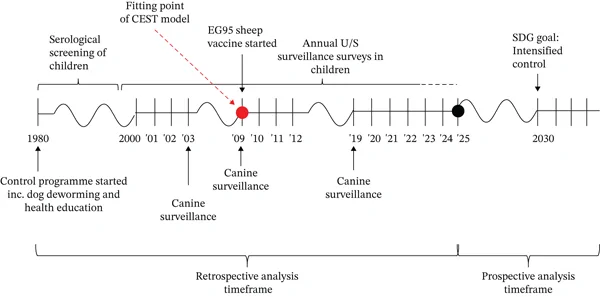
A timeline of historical surveillance and intervention strategies in Río Negro. Dog deworming with praziquantel (PZQ) was started in 1980, as well as serological screening of school children. The first ultrasonographic screening of children (7–13 years) was carried out in 1984, with routine screening in all children (6–14 years) implemented in 1997. Vaccination of sheep with the EG95 vaccine commenced in December 2009 ([30, 31]). The CEST model incorporates all zoonotic host interventions from 1980 until 2025; whereupon, a prospective analysis with different intervention packages is assessed. Abbreviations: U/S, ultrasound; SDG, Sustainable Development Goals.

A mathematical modelling framework, CEST (cystic echinococcosis stochastic transmission) model, was developed to describe the transmission dynamics of CE transmission between dogs and sheep on a typical sheep farm in Río Negro. The characteristics of the model extend upon previously published models for echinococcosis transmission and the wider Taeniidae family, as reviewed by Atkinson et al. 2013 ([28] and Dixon et al. 2019 [29]. Population heterogeneities reflective of the biological processes and husbandry practices of the area were incorporated, as well as control interventions which were implemented in the Río Negro control programme, including dog deworming since 1980, and sheep vaccination since 2009 (Figure 1). A prospective analysis, from 2025 to 2035 was then conducted, in which a set of different intervention scenarios for the intensified control of CE in its zoonotic hosts were evaluated, as per the Sustainable Development Goals for CE.

### 2.1. Model Compartments

The lifecycle of the parasite is reproduced in both the definitive and intermediate hosts, and the environment, via a stochastic population‐based transmission model. Animals from a single farming unit are grouped into compartments representative of their health status at time (*t*). Table 1 provides the variables and parameters used in the CEST model. Animals are age structured in yearly cohorts and progress by discrete monthly timesteps in a probabilistic manner. To capture stochastic variation in transmission, transitions are modelled using binomial draws, with transition probabilities in each monthly timestep derived from an underlying continuous‐time Poisson process. This formulation was chosen because it allows stochastic variation to capture heterogeneity in transmission dynamics whilst retaining a compartmental framework. This provides a natural way to model stochastic event occurrences in continuous time whilst allowing a straightforward translation to discrete monthly probabilities for simulation.

**Table 1:** Notation, definition and units of state variables and parameters included in the CEST model.

**State Variables**		**Description**		

*S*_*d*,*a*_	Susceptible dogs	The number of susceptible animals in age categories (dog years 1, ⋯, 5, and sheep years 1,…,6). The susceptible animals are naive to infection and harbour no parasitic life stages. As they possess no immunity, they can become infected at any time‐point given appropriate exposure.		
*S*_*s*,*a*_	Susceptible sheep			
*E*_*d*,*a*_	Exposed dogs	The number of exposed animals in age categories (dog years 1,…,5, and sheep years 1,…,6). The exposed groups are those animals that have ingested the parasite; however, animals are incapable of onward disease transmission until parasite development has taken place.		
*E*_*s*,*a*_	Exposed sheep	In dogs, this occurs after worm maturation within the intestines and subsequent shedding of eggs, reflective of the prepatent period.		
		In sheep, the time for cyst maturation is unpredictable and highly variable, with not all exposed sheep developing infective cysts within their lifetime. Subsequently, there are three exposed subcategories in which cyst development occurs, for each age group of sheep in the model.		
*I*_*d*,*a*_	Infectious dogs	The number of infectious animals in age categories (dog years 1,…,5, and sheep years 1,…,6). In the infectious compartment, animals are capable of onward disease transmission.		
*I*_*s*,*a*_	Infectious sheep	The sheep population is assumed to be infectious for life.		
*R*_*d*,*a*_	Recovered dogs	The number of recovered dogs in age categories (1,…,5 years). The recovered group contains those animals that are no longer infectious and as they become immune, they do not become reinfected or contribute to transmission (an assumption of full immunity is made).		
*V*_*s*,*a*_	Vaccinated sheep	The number of vaccinated sheep in age categories (1,…,6 years). The vaccinated group are those animals that have been vaccinated, and thus do not further contribute to transmission (an assumption of full protection is made).		
*I*_*e* *g*_	Infectious eggs on pasture	The cumulative number of infectious eggs on the pasture.		

Parameters		Units	Value	Reference

*v*_*d*_	Dog repopulation		Repopulated annually. Set to maintain stable population. New dogs are introduced into the model as young susceptible dogs. The number of dogs repopulated is equal to the number of animals removed the previous year due to natural mortality and the number of retired dogs removed from the population.	Calculated annually
*v*_*s*_	Sheep repopulation		Sheep are repopulated based on the number of culled lambs for meat and removal of old sheep after 6 years. An equal number of new susceptible lambs are introduced into the model based upon the total number of lambs removed and older sheep culled the previous year. This maintains a stable population.	Calculated annually
*μ*_*d*_	Probability of dog dying or being removed from population		10%. A probability of natural mortality is applied randomly to the whole dog population and dogs are subsequently removed from the model regardless of their age or disease status. This is implemented annually.	[32]
*δ*	Average number of eggs shed per infected dog	Month^−1^	250 ^×^550 ^×^2 (based upon documented values of 200–300 worms per dog, producing 500–600 gravid proglottids every 14 days).	([33])
*Ω*	Egg decay probability (non‐viability)		Assuming 2/3 of eggs become non‐viable over 1 year, a minimum 0.02552320 and a maximum of 0.11972445 were calculated (see S1 of Supporting Information).	([34]; [35])
*ε*	Number of cyst development stages (sheep)		3 per age group.	Derived from assuming an Erlang distribution of exposure times
*ƙ*_*d*_	Parasite development rate (dog)	Month^−1^	1/1.5 based on a prepatent period of 1.5 months.	[28]
*ƙ*_*s*_	Cyst development rate (sheep)	Month^−1^	1/4 based on a minimum documented period of 4 months.	([33])
*ϴ*	Culled sheep offal		Proportion of infected offal. Calculated annually from the number of sheep and lambs removed from the population.	Updated annually based on prevalence in sheep
*γ*_*d*_	Dog recovery/retirement from population		1/36 (reciprocal of a mean infectious period in dogs of 3 years). Dogs are assumed to be retired/recovered for the remainder of their life.	
*β*_*d*_	Transmission coefficient dogs	Month^−1^	Contact rate with *i* *n* *f* *e* *c* *t* *e* *d* *s* *h* *e* *e* *p* × *p* *r* *o* *b* *a* *b* *i* *l* *i* *t* *y* of transmission upon contact.	Model fitting
*β*_*s*_	Transmission coefficient sheep	Month^−1^	Contact rate with *i* *n* *f* *e* *c* *t* *i* *o* *u* *s* *e* *g* *g* *s* *x* *p* *r* *o* *b* *a* *b* *i* *l* *i* *t* *y* of transmission upon contact.	Model fitting
*C*_*d*_	Worming effectiveness		The probability of *s* *u* *c* *c* *e* *s* *s* *f* *u* *l* *d* *e* *w* *o* *r* *m* *i* *n* *g* *of* *d* *o* *g* *s* = 65*%* *c* *o* *v* *e* *r* *a* *g* *e* × 100*%* *e* *f* *f* *i* *c* *a* *c* *y*.	([36])
*C*_*s*_	Vaccine effectiveness		The probability of successful vaccination of *s* *h* *e* *e* *p* = 60*%* *c* *o* *v* *e* *r* *a* *g* *e* *of* *f* *a* *r* *m* *s* × 78*%* *e* *f* *f* *i* *c* *a* *c* *y* *p* *e* *r* *d* *o* *s* *e* (98% efficacy given three doses).	([37];[38];[39])

**Figure 2:**
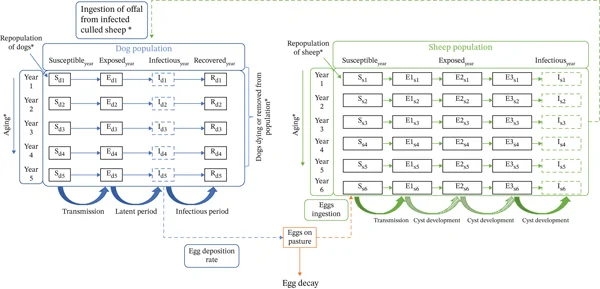
Flow diagram of model. The transmission dynamics of CE on a single farm is illustrated with arrows representing the direction of infection. Worm maturation and subsequent egg deposition on the pasture by infectious dogs (*I*_*d*_), the definitive hosts, results in environmental contamination of the pasture with infectious eggs (*I*_*e* *g*_). The ingestion of eggs by sheep, the intermediate hosts, allows for the onwards transmission of CE. Ingestion of parasite eggs by susceptible sheep leads to an exposed and infectious population of animals (following a period of cyst development), which leads to a potential source of reinfection for dogs when sheep are culled, completing the lifecycle. ∗Yearly rather than monthly event.

Figure 2 presents the flow diagrams in the dog and sheep populations.

### 2.2. Model Dynamics

The model comprises a dog and a sheep population of mixed ages on a single farming unit and explicitly models the annual fluctuations in livestock husbandry seen in Río Negro. When lambs are born into the flock, we assume that 50% are sold for meat at weaning, and 50% are kept as ewe replacements, forming the susceptible first‐year lamb cohort, for a total of 120 sheep in each age cohort. Older animals are culled out after 6 years, when their fecundity and general body condition deteriorates with their declining dentition, hindering their ability to graze efficiently. For *Taenia* species, immunity acquired by the intermediate hosts has been identified as density dependent; however, under natural infection pressure of eggs, sheep do not develop strong immunity [40]. Both prevalence and intensity of infection with CE cysts increase with sheep age [41, 42]; hence, it was assumed that sheep are infected for life. The model is repopulated with a new lamb cohort at the end of each year (every 12th timestep), equal to the number of animals culled out, maintaining a stable population. Of the culled animals, those sheep and lambs that were infected harbour cyst infected offal are a potential source of onwards infection for dogs. Thus, the proportion of infected sheep and lambs within the total culled sheep population informs the risk to the dogs for onward CE infection in the model (*ϴ*).

In Río Negro, most farms have 2–4 dogs; however, the populations are often fluctuating and characterised by high birth and mortality rates [32]. An estimated probability of a dog dying or being retired from the population is applied randomly, at 10% per year, to the entire dog population, regardless of disease status. As the dog population is age stratified, dogs are permanently removed from the population after 5 years. To maintain a stable population size, the model is repopulated based upon the number of animals which have been retired or which have died in the previous year.

Although parasite dynamics are often considered uniformly across different species, heterogeneity in individual animals may result in parasite abundance, as well as different susceptibilities and immunity to infection [28, 43]. In models of *Echinococcus* spp., where these factors are not included, parasite elimination may seem unrealistically easy [28]. There is ongoing discussion regarding the assumption of a flat (equal) force of infection across dog age, with the first model of *E. granulosus* s.l. by Roberts et al. (1986) including age stratification and differing risks per age of the definitive host population. Age‐related parasite load and protective immunity have also been debated extensively in the literature (Roberts et al. 1986; Ming et al. 1992; Torgerson et al. 1998; [44–47]). However, in naturally‐infected dog populations, especially those in high prevalence areas or with a high turnover of dogs, acquired immunity by age is more widely accepted [44, 48]. Differing risk per age group is not explicitly modelled due to the inconclusiveness of the data available; however, the model does include a recovered state for dogs, which animals reach following a period of parasite development and infectiousness (Figure 2). As this is a population‐based model, heterogeneity in the worm burden of dogs cannot be included explicitly; however, the effect it would have in terms of disease dynamics is partially captured by the added overdispersion in egg deposition (see Equation (6)).

Our model framework incorporates an exposed but not yet infectious (or latent) group for dogs, denoted by *E*_*d*_, with parasite development, *k*_*d*_, and rate of progressing to the infectious category 1/*k*_*d*_ reflecting the prepatent period of the parasite inside the dog′s gastrointestinal tract. Afterwards, dogs become infectious and can contaminate the pasture and farming environment with infective eggs, as per the average number of eggs per infected dog parameter (*δ*). Following the dog recovery rate (*γ*_*d*_), dogs are no longer infective, and thus do not further contribute to transmission. They join the recovered group, *R*_*d*_, where they remain until they are aged out of the population.

The dynamics of the number of susceptible dogs (*S*_*d*_) ingesting *E. granulosus* s.l. cysts and becoming exposed dogs (*E*_*d*_); subsequently, becoming infectious (*I*_*d*_), and then recovered (*R*_*d*_), are captured by the following equations. Each equation represents the transitions of animals per time step, (*t*), assuming no intervention measures are in place. Animals move following a probabilistic draw from a binomial distribution, denoted *B* *i*, where *n* is the number of trials (i.e., the number of individuals at that particular disease state), and *p* denotes the probability of success, as shown below:

(1) X~Bin,p.

The probabilistic draw from the binomial distribution is applied separately to each transition between disease states, ensuring individuals leaving one compartment enter the next, conserving the total population numbers.

The subscript *d* and *s* in the model equations are used for dogs and sheep, respectively. The model was age structured, with age categories indexed by *a*. For dogs, *a* = 1, ⋯, 5, whereas for sheep, *a* = 1, ⋯, 6. The subscript letters, *m*, *n*, *o* and *p* pertain to the binomial draw, showing that the number of animals drawn in one equation, are then utilised in the following equation:

(2) Sd,at+1∼Sd,at−BimSd,at,1−exp−βd×ϴ,

(3) Ed,at+1∼Ed,at+BimSd,at,1−exp−βd×ϴ−BinEd,at,1−exp−kd,

(4) Id,at+1∼Id,at+BinEd,at,1−exp−kd−BioId,at,1−exp−yd,

(5) Rd,at+1∼Rd,at+BioId,at,1−exp−yd.

Most CE models assume a homogenous egg distribution within the environment. An aspect that has been underexplored in previous CE models, but that has been considered extensively in other livestock disease models (such as the work of de Cisneros et al. 2014 [49] of sheep infected with nematodes), is the effect of seasonality (temperature and humidity) on egg survival in the environment. Given that environmental egg contamination is how intermediate hosts become infected, this is an integral part of the transmission dynamics in the model.

Experimentally, *E. granulosus* s.l. eggs have been shown to survive extreme temperatures (as low as −50°C for 24 h) with no detectable effects on infectivity [34]. In addition, studies simulating the inferior arid climate of Patagonia, Argentina, (of which Río Negro forms one of six provinces within the region) demonstrated viability and infectiousness of eggs after 41 months exposure [35]. This egg survival time on pasture is important, as a longer duration of viability allows for an increased chance of exposure to infectious eggs by the intermediate host, contributing to the force of infection. A prolonged viability of eggs also contributes to the yearly accumulation of viable eggs, thus increasing the egg density within the environmental and infection pressure. Exact knowledge of egg decay and dispersal within the environment is lacking, in part due to the eggs being morphologically indistinguishable from those of *E. multilocularis* and other taeniids [35]. Consequently, data pertaining to egg accumulation are scarce. Therefore, egg survival studies under experimental conditions, and egg survival rates of other taeniid species were utilised to inform this parameter in the model.

CE eggs within the environment (*I*_*E* *g*_) are subject to an egg decay (non‐viability) probability (*Ω*), reflecting the seasonal weather variations throughout the year, with higher temperatures leading to a greater desiccation of eggs in the summer months (Data in Figure S1, Supporting Information).

The dynamics of the eggs in the environment (*I*_*e* *g*_) are captured in Equation (6). New eggs are introduced into the system from infected dogs of all ages. Eggs are simultaneously removed due to natural decay and non‐viability (*Ω*). The Poisson terms, denoted as *Pois* in the equation, introduce variation in the egg production by the definitive host, acknowledging that egg production and decay are stochastic and not perfectly predictable. The Poisson distribution was used for simplicity, but other distributions could be considered (such as a negative binomial); however, this would have required an additional parameter to be fitted/calibrated, which would have been challenging given the paucity of data available:

(6) Iegt+1∼Iegt+Poisδ×Id,a−PoisΩ×Iegt.

The eggs available for consumption per sheep are calculated as the total number of infectious eggs available in the environment divided by the total sheep population (*I*_*e* *g*_/*N*_*s*_), with the total sheep population denoted as *N*_*s*_ for simplicity.

Once ingested by the sheep, eggs develop into cysts. Viability studies conducted in sheep from Río Negro indicated that 64% of parasitised animals had viable cysts, with the number of viable cysts increasing with age ([42]). The exposed compartment for sheep is modelled using an Erlang distribution with Shape 3 (a type of gamma distribution) in which the category is split into three hypothetical stages (*ε*). The sheep progress through these three stages sequentially according to the parasite development rate, denoted as *k*_*z*_.

When the shape of the Erlang is equal to one, the Erlang distribution reduces to the exponential distribution, which is often not representative of observed biological variation in infectivity, which tends to be more consistent around their mean [50]. Three hypothetical classes are commonly used to add some variation [51], without adding too much computational complexity. This approach has the benefit of reducing the number of sheep that become infected too quickly, allowing for a more gradual transition between parasite stages. As a result, the distribution of cyst development time is more realistic, avoiding spikes in the number of infectious animals due to too fast development time and better capturing the dynamics of infection in sheep. Sheep transition through each Erlang stage with a probability of 1 − exp(−*k*_*s*_ × *ε*), per discrete time step, as shown in Equations (8)–(10).

Afterward, sheep enter the infectious category (*I*_*S*_), where they remain for life. This reflects the chronicity of hydatid cysts and the absence of recovery within their typical production lifespan. When sheep reach 6 years of age, they are removed from the population, regardless of disease status, to reflect the natural lifespan of sheep in the region. These animals, along with half of the lamb population which are also culled, form the culled sheep population (*ϴ*), from which offal is available, posing a risk of infection to the dog population. No natural mortality is applied to the sheep population as there is a paucity of data on this.

The dynamics of the susceptible sheep (*S*_*s*_) becoming exposed to *E. granulosus* s.l. eggs on pasture (*E*_*s*_) and subsequently undergoing cyst development (*I*_*s*_), are captured by the following equations:

(7) Ss,at+1∼Ss,at−BimSs,at,1−exp−βs×Ieg Ns×ε,

(8) Es,at+1~Es,at+BimSs,at,1−exp−βs×IegNs ×ε−BinEs,at,1−exp−ks×ε,

(9) E2s,at+1~E2s,at+BinEs,at,1−exp−ks×ε−BioE2s,at,1−exp−ks×ε,

(10) E3s,at+1~E3s,at+BioE2s,at,1−exp−ks×ε−BipE3s,at,1−exp−ks×ε ,

(11) Is,at+1∼Is,at+BipE3s,at,1−exp−ks×ε.

The transmission coefficients,*β*_*d*_and*β*_*s*_, represent the transmission rates: the contact rate of dogs with infected sheep offal multiplied by the probability of transmission upon contact, and the contact rate of sheep with infectious eggs from the pasture multiplied by the probability of transmission upon contact, respectively. The coefficients were estimated by fitting the model to observed prevalence data obtained from field surveillance data from dogs and sheep, via arecoline purgation and necropsy, respectively, in 2009 in Río Negro prior to the use of the EG95 sheep vaccination ([31]). Model projections were subsequently compared with prevalence estimates from later field surveys that were not used during calibration. Because these surveys differed in the ages of animals assessed and the diagnostic methodologies employed, they were not considered suitable for formal quantitative validation.

Model fitting was done using a regression‐based Approximate Bayesian Computation (ABC) technique [52], as detailed in the *Model Calibration* section of the Supporting Information (Available here). The target prevalences of 4.5% in dogs [53] and 56.3% in adult sheep (> 2 years old) were used ([31, 53]). Where available, model parameters were extracted from published literature, existing CE models and the biological knowledge of *E. granulosus* s.l. and its hosts. The CEST model was coded and run in R [54]. All the code and required data is available and freely accessible in an online repository [55]. Table 1 provides a detailed definition of parameters and initial values. Simulations were initialised using host population sizes representative of a typical sheep farm in Rio Negro, with individuals randomly assigned to disease states, and run to equilibrium before simulations commenced.

The initial starting values for each model compartment are provided in Table S1 of the Supporting Information.

Given uncertainty surrounding the egg decay probability (non‐viability) parameter, we performed a one‐at‐a‐time sensitivity analysis by halving and doubling its baseline value, whereas all other parameters were held constant. Results are shown in Figure S4.

### 2.3. Interventions

The CEST model was used to assess the impact of two different intervention strategies on the risk of disease transmission to humans and zoonotic animal hosts. These included deworming of dogs (*C*_*d*_) with PZQ and vaccination control (*C*_*s*_) of the sheep population with EG95 vaccine (EG95), Table 1.

In the model, deworming of dogs (*C*_*d*_) is applied to the whole dog population; once treated, animals are successfully cleared of parasites and are no longer infectious. PZQ is a very effective taenicide; however, it has no ovicidal effect or residual activity allowing for reinfection. Resultantly dogs in the susceptible (*S*_*d*_), exposed (*E*_*d*_) and infectious compartments (*I*_*d*_) are returned to the susceptible dog population (*S*_*d*_) and are susceptible to CE reinfection. Contrary to this, dogs in the recovered compartment (*R*_*d*_) possess immunity, so remain in the same compartment despite receiving worming treatment.

The vaccine control parameter (*C*_*s*_) is used to inform the proportion of sheep vaccinated with EG95 vaccine and moved to the vaccinated group (*V*_*s*_). It is deemed to be effective if given to susceptible (*S*_*s*_) and exposed animals (*E*_*s*_). However, it is assumed to have no efficacy once an animal is already infected (*I*_*s*_). Sheep vaccination in the model is deterministic, with the number of animals vaccinated based upon the vaccine efficacy and probability of receiving the vaccination, informed by the coverage.

The efficacy of the EG95 vaccination is 98% given a full vaccine course ([37–39]). In Río Negro, lambs are vaccinated in December and January, and then given a further booster dose the following year when they reach 1 year old.

In the model, it was assumed that each of the three doses given has a probability of conferring protective immunity, thus moving individuals to the vaccinated (immune) population (*V*_*s*_). The vaccine efficacy at each time point is adjusted using the following quadratic equation:

(12) 1−x3=10.980.78−,where by x=.

The vaccine programme in Río Negro, commencing in 2009, was limited to indigenous reservations inhabited by the Mapuche population, a subset of the endemic area where the control of the disease presented special geographical and socio‐cultural difficulties [39, 53]. To capture the coverage achieved in Río Negro [39], a baseline vaccine coverage of 60% is assumed in the CEST model. If a farm is not vaccinated, all animals on that farm remain unvaccinated until the next vaccination opportunity.

### 2.4. Scenarios Considered

In our model, the retrospective analysis mimics the historical interventions in Río Negro, with dog deworming implemented from 1980, and sheep vaccination employed since 2009 ([31]). Changes to the control interventions applied are explored in the prospective analysis, covering the period from 2025 to 2035, extending 5 years past the deadline set by the Sustainable Development Goals.

The current regional control programme in Río Negro consisting of sheep vaccination and quarterly dog deworming is used as a counterfactual change nothing scenario (CF) against which alternative intervention packages are compared (Table 2).

**Table 2:** Different intervention scenarios simulated using the CEST model.

Intervention scenario	Dog deworming (PZQ) every x month	EG95 sheep vaccine coverage
Counterfactual (CF)	3	60%
Low frequency (LF)	6	60%
High frequency (HF)	1	60%
Increased vaccine coverage (IC)	3	80%

The WHO veterinary public health core strategic guidelines for PZQ administration in the roadmap for NTD′s are for periodic deworming of dogs; however, no frequency is specified [4]. In the low frequency and high frequency scenario (LF and HF), dog deworming is decreased to twice yearly and increased to monthly, respectively, to understand how the frequency of PZQ administration affects the risk of CE transmission or whether it has a negligible impact. Sheep vaccination remains the same as the status quo.

In the increased vaccine coverage (IC), the frequency of dog deworming is maintained at quarterly administration; however, the coverage of sheep vaccination is increased to 80% as per the WHO′s guidelines for EG95 sheep vaccination [4].

The interventions modelled reflect current policy priorities, require minimal additional investment or infrastructure modification, and are consistent with the WHO NTD roadmap. Although slaughterhouse controls and safe offal disposal have been instrumental in CE control in other settings such as New Zealand ([33]), they were not considered in the CEST model because their effective implementation is currently constrained by weak regulation and the ongoing practice of home slaughter in the Río Negro region.

### 2.5. Model Simulations

The model commences at spring lambing (December in the Southern hemisphere), to coincide with a cohort of new lambs entering the susceptible sheep population. Ten thousand iterations were run for 75 years: 20 years of burn‐in to reach an approximate disease equilibrium, followed by 29 years of deworming of dogs and 16 years of vaccination in the retrospective analysis, and 10 years of alternative intervention packages in the prospective analysis. Each simulation represents a farming unit. All simulations for one scenario are considered as an ensemble with equal weighting.

We extracted the prevalence of CE in both host species at the end of each monthly timestep, considering all ages of dogs and adult sheep (> 1 years) in the calculations. For each scenario, including the counterfactual, we calculated the median prevalence at each timestep, across all simulation runs, along with the corresponding 95% Bayesian credible interval (CI). To summarise annual trends, we then averaged the 12 monthly median prevalence values to obtain a single mean end of year prevalence for each simulation year. Due to the voluntary nature of the vaccine programme resulting in varying intervention uptake across farms, modelled probabilistically based on the vaccine coverage, the 95% CIs reflect both stochastic variation in transmission and differences in intervention uptake between farms. The average proportional change of egg numbers from the counterfactual number of eggs in the environment is also calculated in the same way, representing the risk of disease transmission to humans and zoonotic animal hosts.

## 3. Results

**Figure 3:**
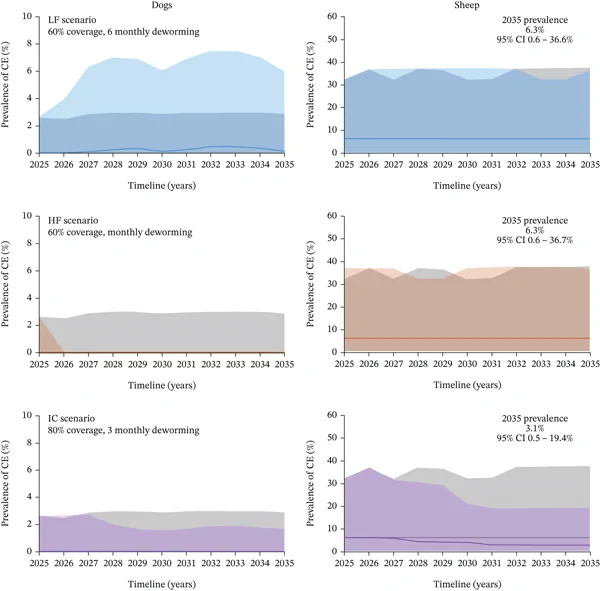
Predicted prevalence of *Echinococcus granulosus* in dogs and sheep under alternative control strategies simulated using the CEST model. The model was fitted to the target prevalences of 4.5% in dogs, 56.3% in adult sheep (> 2 years old), as per surveillance data in Río Negro prior to the onset of sheep vaccination. Projections for 2025–2035 are shown. The grey line and shaded area represent the counterfactual scenario, reflecting continuation of current control activities in Río Negro (60% sheep vaccination coverage and dog deworming every 3 months). Coloured lines and shaded areas represent alternative intervention scenarios: low frequency (LF), 60% sheep vaccination coverage with dog deworming every 6 months; high frequency (HF), 60% sheep vaccination coverage with monthly dog deworming; and increased coverage (IC), 80% sheep vaccination coverage with dog deworming every 3 months. Lines represent annual prevalence estimates obtained by averaging monthly median prevalence values from 10,000 model simulations, and shaded areas represent the corresponding 95% confidence intervals (CI).

The model predicted temporal trends for prospective CE prevalence (2025–2025) based on different intervention packages being implemented are presented in Figure 3. The counterfactual status quo simulation (CF) shows a gradually declining trend in sheep prevalence following implementation of the EG95 vaccination in 2009, as has been seen clinically in Río Negro [53, 56]. The retrospective analysis results are available in Figure S5 of the Supporting Information.

At the end of the retrospective analysis, after 45 years of PZQ and 16 years of EG95 vaccination (year 2025), the model projected prevalence in sheep is 6.31% (CI 95% 0.55–32.63%) which is more optimistic than recent field observations in adult sheep (21.1%) [56]. However, the observed field population consisted of much older animals (> 6 years), which may partly explain the discrepancy.

The predicted prevalence in dogs (0.00%, CI 95%: 0.00–2.61%) is also lower than the most recent surveillance data recorded from ELISA/PCR screening of randomly selected farm dogs in Río Negro in 2017–2018 (prevalence: 8.2%) [53]. No more recent surveillance surveys have been conducted in dogs in the Río Negro region to determine whether the prevalence of CE has remained stable or has continued to decline, as previously observed. At the end of the prospective analysis (year 2035), the sheep prevalence remains consistently low at 6.35%, though some variability is evident as reflected in the wide 95% confidence interval (CI 95%: 0.56%–37.75%). Similarly, the dog prevalence is minimal (0.00%), with occasional fluctuations suggested by the 95% confidence interval (CI 95%: 0.00%–2.86%).

When the frequency of deworming is reduced to 6 monthly, dog prevalence becomes more variable (0.00%, 95% CI: 0.0–5.96); however, the effect on the sheep prevalence is minimal (6.28%, 95% CI: 0.55–36.57%) (LF scenario). This suggests that the control programme could withstand disruptions in dog deworming efforts or a decrease in coverage with minimal short‐term effects on the sheep prevalence providing vaccine coverage is maintained.

In the HF scenario the worming frequency is increased to monthly administration. As expected, this causes a dramatic reduction in the dog prevalence (0.00%, 95% CI: 0.00%–0.00%) and is the only intervention package in which the dog prevalence trends towards transmission disruption. Interestingly, there is only a minimal change to the sheep prevalence (6.34%, 95% CI: 0.55–36.69) suggesting that there is still sufficient environmental contamination of eggs to maintain transmission, and that a lag time exists between good control in dogs and the beneficial effects seen in the intermediate host species.

When the sheep vaccination coverage is increased to 80% (IC scenario) the sheep prevalence drops considerably (3.15%, 95% CI: 0.47%–19.38%), with similar reductions observed in the dog prevalence (0.00%, 95% CI: 0.00%–1.67%). This indicates promising results could be achieved in Río Negro by concentrating efforts on ensuring animals receive a full vaccine course (three doses) and maintaining coverage above 80%.

**Figure 4:**
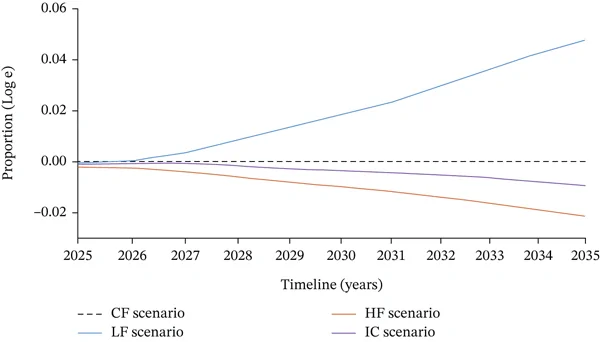
The proportional change (relative to the counterfactual) of *Echinococcus* spp. eggs following the application of different intervention packages. Results are presented as the proportional change in egg numbers (given on a log scale) relative to the counterfactual scenario. Results are presented from the start of the prospective analysis in which new control interventions are implemented. The dotted grey line is the counterfactual. Results rising above this line show an increase in relative egg numbers, as shown in the LF scenario, whereas a decrease from the line represents a decrease in egg numbers, as predicted in the HF and IC scenarios.

The WHO 2030 roadmap to end the neglect to attain the Sustainable Development Goals guidelines for CE are for intensified control. *Echinococcus* spp. eggs in the environment are the source of CE infections in humans and can be regarded as the ‘risk’ for human infections. Thus, controlling and reducing the transmission of the parasite between dogs and sheep will inadvertently reduce this risk to humans, potentially decreasing the number of human cases. The control interventions modelled in the HF and IC scenarios both decrease the egg risk compared with the counterfactual. In the LF scenario, the reduced deworming frequency allows egg deposition by dogs to increase, and subsequently, egg levels in the environment rise. This increases the risk of exposure to humans, Figure 4.

**Figure 5:**
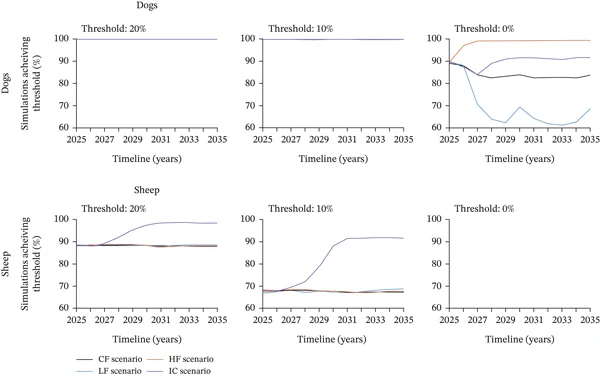
Percentage of stochastic model simulations achieving hypothetical prevalence thresholds in dogs and sheep under alternative control strategies. Results are shown for the prospective intervention period (2025–2035). Lines indicate the proportion of model simulations reaching prevalence thresholds of 20%, 10% and 0% in each host population under each intervention scenario. CF, counterfactual (no change in current control measures, 60% sheep vaccination coverage and dog deworming every 3 months); LF, 60% sheep vaccination coverage with dog deworming every 6 months; HF, 60% sheep vaccination coverage with dog deworming every month and IC, 80% sheep vaccination coverage with dog deworming every 3 months.

The target for CE set by the WHO in its roadmap is for intensified control; however, this is not quantified further by a specific disease target. We set three hypothetical prevalence thresholds (20%, 10% and 0%) for dogs and sheep, with the aim of understanding which control packages may lead to transmission disruption within our prospective analysis time frame (Figure 5).

At the end of the prospective analysis timeframe, 87.79% of the simulations from the CF scenario, 88.82% from the LF, 88.34% in the HF scenarios and 98.43% in the IC scenario, reached the imposed 20% disease threshold in sheep. Considering the 10% disease threshold, the proportion of simulations reaching this level decreased to 67.47% for CF, 68.71 for LF, 67.70 for HF scenarios and 91.6% of simulations from the IC scenario. Notably, none of the CEST model runs reached CE transmission disruption in sheep, defined by the 0.0% prevalence threshold.

The prospective analysis trends for dogs are broadly similar, with 100% of simulations in all scenarios reaching the 20% disease threshold, and 99% of simulations from all the scenarios reaching the 10% disease threshold. Considering the theoretical threshold of transmission disruption (0.0%), the HF scenario, in which deworming is administered monthly, is a faster method of achieving transmission disruption than increasing vaccination coverage with 99.35% of simulations reaching this target in dogs compared with 91.70% in the IC scenario. However, the difference between the two strategies is minimal.

## 4. Discussion

The CEST model captures and replicates the disease dynamics of CE in the Río Negro region since the onset of the control programme in the 1980s. Furthermore, it can be used as a predictive tool to explore the impact of different control strategies which may accelerate the progress towards enhanced control of CE, as targeted by the Sustainable Development Goals.

Overall, our simulations are broadly similar. The HF scenario emerges as the most rapid intervention for reducing dog prevalence, which also translates to the greatest reduction in E*.granulosus* s.l. eggs, although this reduction is only modest. The IC scenario, although slower in reducing dog prevalence, also has the added advantage of significantly reducing sheep prevalence too, making it a potentially more attractive control strategy depending on the programme′s primary objective. For instance, it may be more favourable from an animal health and economic standpoint, whereas the HF scenario may be preferred when prioritising a reduction in environmental egg burden and human CE risk.

Within the prospective timeframe modelled, only the HF scenario shows a clear trajectory towards transmission disruption in dogs, with a subsequent reduction in eggs in the environment also.

In the model simulations, dog dosing with PZQ had minimal impact on sheep prevalence, regardless of dosing frequency. This has also been observed clinically in control programmes in the Río Negro region of Argentina and the Middle Atlas Mountain range in Morocco ([36, 57]). In Río Negro, although sheep prevalence initially declined after PZQ was introduced to dogs in the 1980s, fluctuations in prevalence persisted, indicating ongoing transmission ([36]). Similarly, in Morocco, no significant effect of dog treatment was observed on the incidence of *E. granulosus* in sheep or the abundance of viable cysts [57]. This limited impact is likely due to lambs being born into heavily contaminated environments, leading to early exposure of *E.granulosus* s.l. eggs, and subsequent infection during early life. The continual availability of eggs to sheep in the model also likely amplifies the transmission between species, despite the negative feedback of non‐viability and seasonal decay applied to the egg population in the model.

Halving and doubling the egg decay probability parameter produced only minor changes in predicted host prevalence, indicating that model outputs were relatively insensitive to uncertainty in this parameter. This limited influence is likely due to the delayed progression of infection in sheep, represented by the multistage exposed compartments, which buffer transmission dynamics and dampen the effects of variation in environmental egg availability.

When vaccination coverage was modelled at 60% (LF and HF scenarios), vaccination appeared less effective compared with the IC scenario, where coverage was increased to 80%. This highlights the critical role of coverage in determining the success of vaccination efforts and underscores the importance of ensuring that all animals receive the full three‐dose vaccine course and that the higher coverage of animals is achieved. Achieving higher coverage should be a priority, and a cost‐effectiveness analysis is recommended to assess the economic impact of this strategy compared with the other intervention scenarios.

Human CE cases are still being diagnosed in the Río Negro region, with a constant exposure though a rural lifestyle and the disease being acquired in childhood recognised as high risk factors [58]. However, the link between livestock infections and human infections is poorly understood [59]. Consequently, in our analyses we quantified human infection risk using the environmental burden o*f E. granulosus* eggs as a proxy, as human exposure occurs through accidental ingestion of eggs shed by infected dogs rather than through direct contact with infected livestock. Due to the paucity of information and imprecision of egg identification under field conditions, we did not fit the environmental egg numbers in the model. We deliberately only report the proportional change in egg numbers (relative to the counterfactual) rather than exact egg numbers from our simulations. There are many factors regarding the survival of eggs in the environment which are poorly understood. Under experimental conditions, egg survival [34], and egg viability and infectiousness of eggs to sheep experimentally infected in Patagonia are documented [35]. However, the changing climate and weather patterns on egg survival and dispersal since 2005 have yet to be studied for *E. granulosus* s.l. eggs. In 2011, the Puyehue‐Cordón Caulle volcanic complex erupted covering agricultural land between Jacobacci and Bariloche (townships within the Río Negro province) with up to 5 cm of fine ash [60]. Prior to this, there had been very little rainfall in the region, with years of drought. The compounding effects of the ash and previous reductions in vegetation growth led to livestock losses due to starvation, dehydration and poor dentition due to tooth abrasions. The volcanic ash also led to a decline in birth rates (from 60% to circa 10%–30%) as ewes were stressed and malnourished [60]. The effects of ash on CE egg survival and dispersal are however unknown. Although the model incorporates heterogeneous egg viability by month, it does not capture extreme weather events which may affect egg survival and disease transmission risk.

In our model, we reproduce the husbandry practices of the Río Negro province, including the annual culling of older sheep and lamb replacement into the flock. Another poorly quantified effect of the volcanic eruption is the changing dynamics of the flock; without the birth of replacement lambs, the sheep population is not being replenished with younger, local animals. This would lead to an older population of sheep, with a potentially higher burden of CE infected cysts. Both these factors could have profound consequences on the disease transmission dynamics in Río Negro and may explain why our model predictions are lower than those most recently documented in the field by Labanchi et al. [56]. Updated surveillance data in both dogs and sheep are essential to be able to monitor the progress of the existing CE control programme and cross‐validate the prospective analysis model outputs in real time.

Sheep vaccination was heralded as the holy grail of CE disease control when it was first introduced [16, 61], especially to reduce the biomass of cysts in sheep and subsequent infection pressure to dogs ([33]). However, despite 16 years of annual sheep vaccination in Río Negro, a small number of CE cases are still being detected in children and adults in the region (human prevalence 0.20% [95% CI: 0.1–0.3]) [53], indicating that transmission has not been completely interrupted. Consistent with these observations, our model predicts substantial reductions in infection prevalence but not elimination. The wide confidence intervals around model estimates further highlight the heterogeneity in transmission dynamics and intervention uptake across farms, suggesting that although sheep vaccination is an important component of control, additional factors are likely contributing to the persistence of transmission and may require complementary control measures.

In addition to the intervention scenarios explored in the prospective analysis, adjuncts to existing control measures that may help mitigate disease transmission further could include the introduction of communal slaughter facilities with appropriate offal disposal, education and more stringent measures and incentives to prevent unlawful home slaughtering. In Río Negro, home slaughtering of sheep and the feeding of offal to dogs is commonplace due to the lack of slaughter facilities in the region and prohibitive cost of commercial dog food. In countries that have eliminated CE, like New Zealand and Iceland, education and the strict banning of home slaughter, were instrumental to their success ([33]). The Icelandic CE control campaign was exclusively based on education for the first 20 years, with enforcements to sheep husbandry, slaughter and dog husbandry implemented in the generations thereafter. In New Zealand, following a slightly less impactful education campaign, a law banning offal feeding to dogs, a taxation to dog owners, and mandatory dog deworming was introduced. Slaughterhouses were also built across the country, and it was made illegal to slaughter animals outside of them ([33, 62]). Alternative options to enhance CE control in dogs, negating the need for regular anthelmintic treatments and good owner compliance, is the use of slow release PZQ preparations (subcutaneously implanted into dogs and experimentally into mice), which have been demonstrated to block reinfection for up to 2 years [63, 64]. Historically resistance from farmers and high costs made this option unsuitable for use in Argentina. However, given PZQ′s effectiveness as a taenicide, it could be reconsidered with the aim of reducing disease risk to both livestock and humans. Wildlife species are also not considered part of the transmission dynamics of CE in Río Negro; however, with wild carnivores and herbivores present, this is an underexplored area, which may be thwarting control efforts in the region.

A limitation of this study is the absence of a formal independent validation dataset, reflecting the limited surveillance data available for CE. Although model projections were compared with prevalence estimates from later field surveys, differences in the ages of animals sampled and the diagnostic methodologies employed precluded direct quantitative validation. Nevertheless, the broad agreement between projected and observed prevalence trends provides support for the model′s ability to capture the underlying transmission dynamics.

A further limitation relates to the exploration of parameter uncertainty and sensitivity. Parameter uncertainty was incorporated through Bayesian calibration and propagated across simulations, with 95% CIs used to reflect variability in model outputs. In addition, targeted scenario analyses were conducted to assess sensitivity to key intervention parameters, including variation in sheep vaccination coverage (60%–80%) and dog deworming frequency (twice yearly to monthly) in line with WHO guidance. These analyses provide policy relevant insight into the sensitivity of model outcomes to key control strategies. However, a formal global sensitivity analysis (e.g., variance‐based methods) was not performed, and future work should aim to systematically quantify parameter influence and interactions across the full parameter space.

Together, these approaches allow uncertainty and key intervention sensitivities to be explored without over‐parameterising the model in the absence of supporting data.

Our model predictions may differ from real world surveillance data due to certain assumptions including the exclusion of parasite overdispersion in dogs and sheep, and the omission of anomalous husbandry practices, specifically, the feeding of infected sheep offal to dogs. The parameter for culled sheep offal (*θ*) is calculated annually based on the number of sheep and lambs removed from the model population. However, if livestock die or are slaughtered throughout the year and their offal becomes available to dogs, this could increase parasite transmission to dogs, potentially resulting in a higher prevalence than predicted by our model. Although home slaughtering and offal feeding are prohibited, they are likely to still occur on occasion in the region. This may partly explain why our model predictions are lower than recent surveillance data and incorporating more stochasticity in this parameter could help align our model outputs more closely to real‐world observations.

Although our model does not include a spatial component to it, with the effect of ‘super‐spreader’ hosts being counteracted by a refugia of parasite eggs in the environment, it would be interesting to compare the results of an individual based model of echinococcosis with the outputs of the CEST model to establish the impact of these factors on model outputs.

Additionally, a few simplifications are made in the CEST model design which may not fully capture the complexity of CE transmission and its control in Río Negro. We assume that a farm is worming its dogs regardless of its sheep vaccination status. Although this could be true, it is optimistic to assume compliance from a producer for one aspect of the control programme whilst declining another part of it. We also assume regular deworming treatments of dogs, as per the frequency schedule intended. When worming tablets are administered to dogs by veterinary staff, correct dosing is most likely executed; however, when tablets are dispensed to the owner to be administered later under their own responsibility, there is potential for uncertainty. The dog may not receive the dose, or the frequency prescribed might not be adhered to. This heterogeneity is not currently captured in the CEST model and may account for some of the transmission still occurring in the region despite regular anthelmintic treatments documented since the 1980s.

## 5. Conclusions and Future Work

CE causes an economic and public health burden to affected countries. Given its complex zoonotic transmission and NTD status, CE is a priority for human health, social care and animal health practitioners, and will almost certainly require proactivity from all parties to reduce the disease burden further. The WHO 2030 roadmap to end the neglect to attain the Sustainable Development Goals is fast approaching. The roadmap target for CE is for intensified control, with critical action highlighted in the areas of planning, governance and programme management, monitoring and evaluation and access and logistics identified [4]. Clearer and more specific disease control targets for CE in the roadmap would be beneficial. As this study demonstrates, the likelihood of meeting these targets depends heavily on how thresholds are defined. To support a proactive approach, these targets should be more precisely articulated at the policy level.

The CEST model is a valuable tool to help meet some of the roadmap targets. Although there is a successful control programme implemented in the region, there is a risk that the programme could be discontinued or lose necessary funding given a paucity of surveillance data to demonstrate its effectiveness and the long‐term cost requirement of the programme.

Our analyses indicate that additional intervention intensity is required to break the current status quo and further intensify disease control.

Given additional input into its model framework and parameters, the CEST model has the capability of being extended to include additional control measures and simulate their likely effectiveness under different intervention scenarios. These scenarios could involve improved community engagement, a reduction in home slaughtering, and the cost‐effectiveness of various control strategies. Additionally, the model could incorporate other host species, such as goats, and consider the role of wildlife reservoirs, which currently remain poorly understood. With stakeholder engagement and a cost‐benefit analysis of different intervention packages, the model serves as a valuable tool to aid policymakers and stakeholders in future CE control decisions.

**Nomenclature**
ABC: Approximate Bayesian Computation
CE: cystic echinococcosis
CEST model: cystic echinococcosis stochastic transmission model
DALYs: disability‐adjusted life years
EG95: sheep vaccination against echinococcosis
GBD: Global Burden of Disease
NTD: neglected tropical disease
PZQ: praziquantel
WHO: World Health Organization
zNTD: zoonotic neglected tropical disease

## Author Contributions

Conceptualisation: Jo Widdicombe, Daniel Jackson, María‐Gloria Basáñez, Edmundo J. Larrieu, Guillermo Mujica, Mahbod Entezami and Joaquín M. Prada. Formal analysis: Jo Widdicombe. Funding acquisition: María‐Gloria Basáñez, Daniel Jackson, Edmundo J. Larrieu and Joaquín M. Prada. Investigation: Jo Widdicombe, Edmundo J. Larrieu, Guillermo Mujica and Joaquín M. Prada. Methodology: Jo Widdicombe, Daniel Jackson, María‐Gloria Basáñez, Edmundo J. Larrieu and Joaquín M. Prada. Project administration: Jo Widdicombe, María‐Gloria Basáñez, Edmundo J. Larrieu, Guillermo Mujica and Joaquín M. Prada. Supervision: Daniel Jackson, María‐Gloria Basáñez, Edmundo J. Larrieu and Joaquín M. Prada. Writing—original draft preparation: Jo Widdicombe. Writing—review and editing: Jo Widdicombe, Daniel Jackson, María‐Gloria Basáñez, Edmundo J. Larrieu, Guillermo Mujica, Mahbod Entezami and Joaquín M. Prada.

## Funding

This study was supported by the Doctoral college studentship, University of Surrey, European Partnership on Animal Health & Welfare (PAHW) (101136346), One Health European Joint Project (773830) and Medical Research Council Centre for global infectious disease analysis (MR/R015600/1).

## Ethics Statement

Ethical approval was sought from the University of Surrey′s ethical committee. Ethics approval was not required as this study contains no human participants. All data used was freely available in the published literature.

## Conflicts of Interest

The authors declare no conflicts of interest.

## Supporting information


**Supporting information** Additional supporting information can be found online in the Supporting Information section. Supporting information on the parameterisation and fitting of the CEST model is available in the accompanying Supporting Information. Figure S1: Seasonal egg viability. Figure S2: Model fitting utilising MCMC techniques. Figure S3: Final prevalence values of dogs and sheep obtained from the fitting process. Figure S4: Sensitivity analysis of the egg decay probability (non‐viability) parameter. Figure S5: Predicted prevalence of *Echinococcus granulosus* in dogs and sheep under alternative control strategies simulated using the CEST model. Table S1: Egg decay probability (non‐viability) by month. Table S2: Initial starting values of animals in each model compartment. Table S3: Prior values and target model outputs for dogs and sheep after 64 years of simulations for each particle.

## Data Availability

The authors confirm that the data supporting the findings of this study are available within the article [and/or] its Supporting Information. The code for the analysis is publicly available at https://github.com/JoWidds/Cystic-echinococcosis-stochastic-transmission-CEST-model.
