# Supplementary material for: Modelling the Transmission Dynamics and Risk of Ovine Cystic Echinococcosis in Argentina Using the CEST Model (Cystic Echinococcosis Stochastic Transmission Model)
Source: J Parasitol Res. 2026 Sep 10;2026:6394607. doi: 10.1155/japr/6394607 (PMC13560500; doi:10.1155/japr/6394607)
Supplement: Supplementary file 1 [file JAPR-2026-6394607-s001.docx]

**Supporting information**

Widdicombe *et al.* 2026

Index

Egg viability

Initial starting values of animals

Model calibration

Sensitivity analysis of the egg decay probability (non-viability) parameter

Results extended

Bibliography

List of figures.

Figure S1. Seasonal egg viability

Figure S2. Model fitting utilising MCMC techniques

Figure S3. Final prevalence values of dogs and sheep obtained from the fitting process.

Figure S4. Sensitivity analysis of the egg decay probability (non-viability) parameter

Figure S5. Predicted prevalence of *Echinococcus granulosus* in dogs and sheep under alternative control strategies simulated using the CEST model.

List of tables.

Table S1. Egg decay probability (non-viability) by month

Table S2. Initial starting values of animals in each model compartments

Table S3. Prior values and target model outputs for dogs and sheep after 64 years of simulations for each particle.

**Egg viability**

Cystic echinococcosis (CE) eggs are very resistant to environmental conditions such as extreme cold, however they are sensitive to desiccation. Higher temperatures in the summer months lead to a greater desiccation of eggs, with 2/3rds of eggs destroyed over 1 year, with peak decay occurring at the height of summer. In the model, this is reflected by the egg decay rate (*Ω*) which is applied to the total egg population within the environment (I*_Eg_*) at each monthly timestep (*t*), Figure S1, Table S1.

Figure S1. Seasonal egg viability. A probability for the desiccation of eggs at each monthly timestep is applied in the CEST model, providing seasonal variation and negative feedback to the total number of eggs in the model. The parameter is calculated using a sinusoidal wave with the equation 0.11*sin(x/4)+ 0.01 for each timepoint. Peak egg decay occurs at the height of the summer.

Table S1. Egg decay probability (non-viability) by month

| Month | Value |
| --- | --- |
| 1 | 0.03721444 |
| 2 | 0.06273681 |
| 3 | 0.08498026 |
| 4 | 0.10256181 |
| 5 | 0.11438831 |
| 6 | 0.11972445 |
| 7 | 0.11823845 |
| 8 | 0.11002272 |
| 9 | 0.09558805 |
| 10 | 0.07583194 |
| 11 | 0.05198271 |
| 12 | 0.02552320 |

**Initial starting values of animals**

Each simulation of the CEST model represents an individual farming unit. The number of animals on a typical sheep farm in Rio Negro are used to populate the initial model compartments, Table S2. Animals are arbitrarily assigned a disease status, and the model is run for 20 years prior to any interventions to reach a disease equilibrium between the different populations. For the simulations the population sizes were scaled by a factor of 10.

Table S2. Initial starting values of animals in each model compartments. Animals age through the model, with the dog and sheep population being replenished after 5 and 6 years respectively.

|  | Model variables | Initial values | | | | | |
| --- | --- | --- | --- | --- | --- | --- | --- |
|  |  | Year 1 | Year 2 | Year 3 | Year 4 | Year 5 | Year 6 |
| S_d_ | Susceptible dogs | 0 | 0 | 0 | 0 | 0 | - |
| E_d_ | Exposed dogs | 2 | 0 | 0 | 1 | 0 | - |
| I_d_ | Infectious dogs | 0 | 1 | 0 | 1 | 1 | - |
| R_d_ | Recovered dogs | 0 | 0 | 0 | 0 | 1 | - |
| S_s_ | Susceptible sheep | 40 | 20 | 20 | 20 | 20 | 20 |
| E_s1_ | Exposed sheep (group 1) | 40 | 20 | 20 | 20 | 20 | 20 |
| E_s2_ | Exposed sheep (group 2) | 40 | 20 | 20 | 20 | 20 | 20 |
| E_s3_ | Exposed sheep (group 3) | 40 | 20 | 20 | 20 | 20 | 20 |
| I_s_ | Infectious sheep | 40 | 20 | 20 | 20 | 20 | 20 |
| I_eg_ | Eggs on pasture | (250*550*2)*12*7  (egg deposition (δ))* 12 (months) * ∑ no. dogs | | | | |  |

**Model Calibration**

The model is calibrated to dog and sheep prevalence values in 2009, prior to the introduction of the EG95 vaccine in sheep. We adapted and modified the methods of Beaumont *et al.* 2010 and Prada *et al*. 2014 to calibrate the model (Prada Jiménez de Cisneros et al. 2014; Beaumont 2010) to estimate the posterior distribution of the beta transmission rates (β_d_ and β_s_) using Bayesian inference. The model was initialised with prior parameter values for each particle (dogs and sheep) generated by randomly sampling, with replacement, from a uniform prior distribution range, Table S3. 10,000 parameter vector were generated, and the model was run for 20 years with no interventions (allowing it to reach a pseudo equilibrium), and then for a further 29 years with quarterly praziquantel administration to the dogs. Generated model outputs of prevalence in dogs, lambs and sheep were then recorded at the end of the simulations, melded alongside the corresponding beta value used to generate them.

Table S3. Prior values and target model outputs for dogs and sheep after 64 years of simulations for each particle.

|  | Dogs | Sheep |
| --- | --- | --- |
| Priors (β_d_ and β_s_) | random uniform distribution between 3 and 6 | random uniform distribution between 0.9 and 1 |
| Target prevalence | 4.5% | 56.3% in adult sheep (> 2 years old) |

The first step for the calibration was an ABC–rejection algorithm. The explicit aim was the target prevalence of 4.5% in dogs and 56.3% in sheep, as per field surveillance data obtained from dogs and sheep, via arecoline purgation and necropsy respectively, in Río Negro prior to the introduction of the EG95 vaccine in the Rio Negro control programme (Larrieu et al. 2013; Mujica et al. 2021). The empirical standard deviation for each particle was calculated to standardise the data, with parameters from each iteration then accepted or rejected based on a given tolerance level, with the 0.1% closest values to the target prevalences retained (1000 values).

To account for the error between the accepted and observed summary statistics, where a smaller error would mean a better fit, a regression was carried out to further improve the estimation of the posteriors. Accepted simulations, which more closely matched the observed summary statistics, are given more weight by assigning a distance kernel, as in Beaumont *et al.* 2010.The accepted particles are then weighed, (i.e. their position adjusted in parameter space) and then those points which have a nonzero weight have a linear regression applied to them to obtain an estimate. Negative values are removed from the data, and then the corrected particles are taken to be a random draw from an approximation of the posterior distribution. These fitted values are then fed back into the model again and the process is repeated a further 5 times, akin to a Sequential Monte Carlo approach (SMC). The results of this process are the generation of particles which progressively converge around the target prevalence values, Figure S2 and Figure S3. The final ABC algorithm and corrected particles yields an approximate posterior distribution for each of the transmission coefficients, β_d_ and β_s_ which are subsequently employed, through random draws, in our model simulations.

Figure S2. Model fitting utilising MCMC techniques estimate the posterior distribution of the β_d_ and β_s_ transmission coefficients from the given prior distribution. The model simulations and fitting process was repeated 5 times, generating particles which optimally converged to observed prevalence data in dogs after 29 years of PZQ administration and adult sheep in Río Negro prior to the introduction of the vaccination (Larrieu et al. 2013; Mujica et al. 2021).

Figure S3. Final prevalence values of dogs and sheep obtained from the fitting process. Each point represents the outcome of a simulation using a unique combination of βd and βs transmission coefficients. Horizontal and vertical lines indicate the target prevalence values used for fitting.

**Sensitivity analysis of the egg decay probability (non-viability) parameter**

Egg numbers were not fitted in the model due to the paucity of data on environmental egg numbers, and lack of differentiation possible between *taeniae* species. Given uncertainty in the egg decay probability (non-viability) parameter, we performed a one at a time sensitivity analysis in which the parameter was reduced to half and increased to twice its baseline value (1000 simulations), while all other parameters were held constant, Figure S4.

Figure S4. Sensitivity analysis of the egg decay probability (non-viability) parameter. The egg decay probability (non-viability) was varied in a one at a time sensitivity analysis by reducing the baseline value by 50% and increasing it by 100% (2× baseline), while all other model parameters were held constant. Baseline model predictions are shown alongside the reduced and increased parameter scenarios for comparison. Changes in the egg decay probability (non-viability) produced only minor effects on predicted host prevalence, indicating that model outputs are relatively insensitive to uncertainty in this parameter.

**Results – extended**

Figure S5. Predicted prevalence of *Echinococcus granulosus* in dogs and sheep under alternative control strategies simulated using the CEST model. The model was fitted to the target prevalences of 4.5% in dogs, 56.3% in adult sheep (> 2 years old), as per surveillance data in Río Negro prior to the onset of sheep vaccination. Projections for 2009–2035 are shown. The dotted vertical line is the start of the prospective analysis (year 2025 – 2035). The grey line and shaded area represent the counterfactual scenario, reflecting continuation of current control activities in Río Negro (60% sheep vaccination coverage and dog deworming every 3 months). Coloured lines and shaded areas represent alternative intervention scenarios: Low Frequency (LF), 60% sheep vaccination coverage with dog deworming every 6 months; High Frequency (HF), 60% sheep vaccination coverage with monthly dog deworming; and Increased Coverage (IC), 80% sheep vaccination coverage with dog deworming every 3 months. Lines represent annual prevalence estimates obtained by averaging monthly median prevalence values from 10,000 model simulations, and shaded areas represent the corresponding 95% confidence intervals (CI).

**Bibliography**

Beaumont, Mark A. 2010. ‘Approximate Bayesian Computation in Evolution and Ecology’. *Annual Review of Ecology, Evolution, and Systematics* 41 (1): 379–406. https://doi.org/10.1146/annurev-ecolsys-102209-144621.

Larrieu, Edmundo, Eduardo Herrero, Guillermo Mujica, et al. 2013. ‘Pilot Field Trial of the EG95 Vaccine against Ovine Cystic Echinococcosis in Rio Negro, Argentina: Early Impact and Preliminary Data’. *Acta Tropica* 127 (2): 143–51. https://doi.org/10.1016/j.actatropica.2013.04.009.

Mujica, Guillermo, Leonardo Uchiumi, Daniel Araya, et al. 2021. ‘The Diagnosis, Treatment, Surveillance and Control of Cystic Echinococcosis in the Province of Rio Negro: The “One-Health” Model’. *Parasitologia* 1 (4): 4. https://doi.org/10.3390/parasitologia1040019.

Prada Jiménez de Cisneros, Joaquín, Michael J. Stear, Colette Mair, et al. 2014. ‘An Explicit Immunogenetic Model of Gastrointestinal Nematode Infection in Sheep’. *Journal of The Royal Society Interface* 11 (99): 20140416. https://doi.org/10.1098/rsif.2014.0416.
